# Painless Visible Haematuria in Adults: An Algorithmic Approach Guiding Management

**DOI:** 10.7759/cureus.6140

**Published:** 2019-11-13

**Authors:** Gurjeet Dulku, Arjun Shivananda, Aron Chakera, Richard Mendelson, Dickon Hayne

**Affiliations:** 1 Radiology, Royal Perth Hospital, Perth, AUS; 2 Radiology, Sir Charles Gairdner Hospital, Perth, AUS; 3 Nephrology, Perth Children's Hospital, Perth, AUS; 4 Urology, University of Western Australia School of Medicine, Perth, AUS

**Keywords:** urology, hematuria, painless hematuria, imaging, radiology, radiology

## Abstract

There is consensus that visible haematuria may be a sign of serious underlying disease, including malignancy, and warrants a thorough diagnostic evaluation. This is usually undertaken by a combination of clinical examination, cystoscopic evaluation, and urinary tract imaging.

A decision support tool has been developed in the form of an algorithmic flow chart as part of a suite of on-line evidence-based and consensus-based guidelines Diagnostic Imaging Pathways (DIP): www.imagingpathways.health.wa.gov.au (Online clinical decision-making tool: Dulku G. Painless Macroscopic Haematuria. Diagnostic Imaging Pathways; September 2015) to provide imaging recommendations for adult patients with unexplained, painless visible haematuria. A literature review, including reference to several international consensus-based expert guidelines, has been employed to develop this tool.

The choice of first line imaging method is dependent on the risk stratification into high or low risk for the development of renal and urologic malignancies. Ultrasound is vital in the initial assessment of haematuria particularly in radiation sensitive patients, low-risk patients, and in young men <40 years. Computed tomographic urography (CTU) is a sensitive and specific method for the detection of urothelial malignancy particularly in high-risk patients. Magnetic resonance urography (MRU) provides better contrast resolution than CTU without exposure to ionising radiation or requiring intravenous (IV) contrast administration, making it more suitable for examination of paediatric and pregnant patients and patients with renal impairment. Cystoscopy remains the gold standard in the detection of lower urinary tract (bladder) urothelial tumours.

Until randomised clinical trials comparing different diagnostic modalities or strategies prospectively and outcome studies are available, consensus-based practice recommendations similar to ours are nonetheless warranted to reduce the variation in haematuria management.

## Introduction and background

The presence of haematuria may be the sole symptom of an underlying disease, either benign or malignant. It is one of the most common presentations of patients with urinary tract diseases and of patients referred for urinary imaging. Painless visible haematuria (VH) is the commonest presentation of bladder cancer.

The prevalence of urological malignancy among patients with VH has been reported to be as high as 19-24% but more typically ranges between 3% and 6% [1-7]. Thus, VH warrants a thorough diagnostic evaluation, and this is usually done with a combination of clinical examination, cystoscopic evaluation, and urinary tract imaging [4, 8-11].

The diagnosis is often delayed due to the similarity of these symptoms to benign disorders (e.g., urinary tract infection, interstitial cystitis, prostatitis, passage of renal calculi), and delays can lead to a worsened prognosis due to more advanced stage at diagnosis [10].

Bladder tumours account for 90-95% of urothelial carcinomas (UCs) and are the most common urinary tract malignancy. The most common symptom of bladder cancer is haematuria, which usually occurs suddenly and is generally painless [12]. In 2011 alone, 2404 new cases of bladder cancer were diagnosed in Australia. It is significantly more common in men, with the risk of bladder cancer by the age of 85 is one in 43 for men compared to one in 166 for women. In 2012, there were 1038 deaths caused by bladder cancer in Australia. Other less common symptoms include incomplete voiding, dysuria and frequency which also may be seen with other benign conditions. The five-year survival rate for Australians with invasive bladder cancer is 58% [13].

Low-risk factors for the development of urothelial or bladder cancer include, all patients under the age of 40 years without a history of smoking, family history of urothelial or bladder cancer or exposure to urothelial carcinogens. On the other hand, high-risk factors for the development of renal and urologic malignancies among patients with VH include male patients, particularly those with a smoking history or family history or occupational exposure to urothelial carcinogens. Although the risk is still small in men <40 years of age, whether to place such patients in low- or high-risk categories is discretionary and should be based on clinical features and presence of other risk factors. The risk factors for the development of renal or urologic malignancies are listed in Table 1 [2, 14, 15].

**Table 1:** Risk factors

Risk factors for the development of renal and urologic malignancies
Males
Increasing age
Smoking
Occupational exposure to urothelial carcinogens, e.g. metal workers, painters, and rubber manufacture
Family history
Pelvic irradiation
Chronic inflammation of urinary tract, e.g. calculus, diverticula, infection, and analgesic abuse (e.g., phenacetin)

Currently, there is insufficient data available to derive a rigorously evidence-based algorithm of the diagnostic pathway for haematuria. An algorithm based on the best available evidence, a consensus of clinical experts in the review team, other published guidelines and the results of economic modelling is currently being utilised as guidance [16].

## Review

Methodology

An electronic search from August-September 2015 of MEDLINE through PubMed, and the Cochrane Database of Systematic reviews identified relevant original articles, systematic reviews and evidence-based guidelines from the period of 2008-September 2015, which were included. The following terms were used in combination during literature review to produce the list of articles reviewed: “painless”, “macroscopic”, “visible” AND “haematuria”, “kidneys”, “ureters”, “urinary bladder”, “bladder cancer”, “urinary tract”, “cytology”, “diagnostic imaging”, “radiography”, “ultrasonography”, “ultrasound”, “pyelography”, “tomography, X-ray computed” AND “urography”, “magnetic resonance” AND “urography”, “intravenous” OR “excretory” AND “urography”, “urology” OR “nephrology” AND “referral” OR “consultation”, “cystoscopy”, “guidelines”.

**Figure 1:**
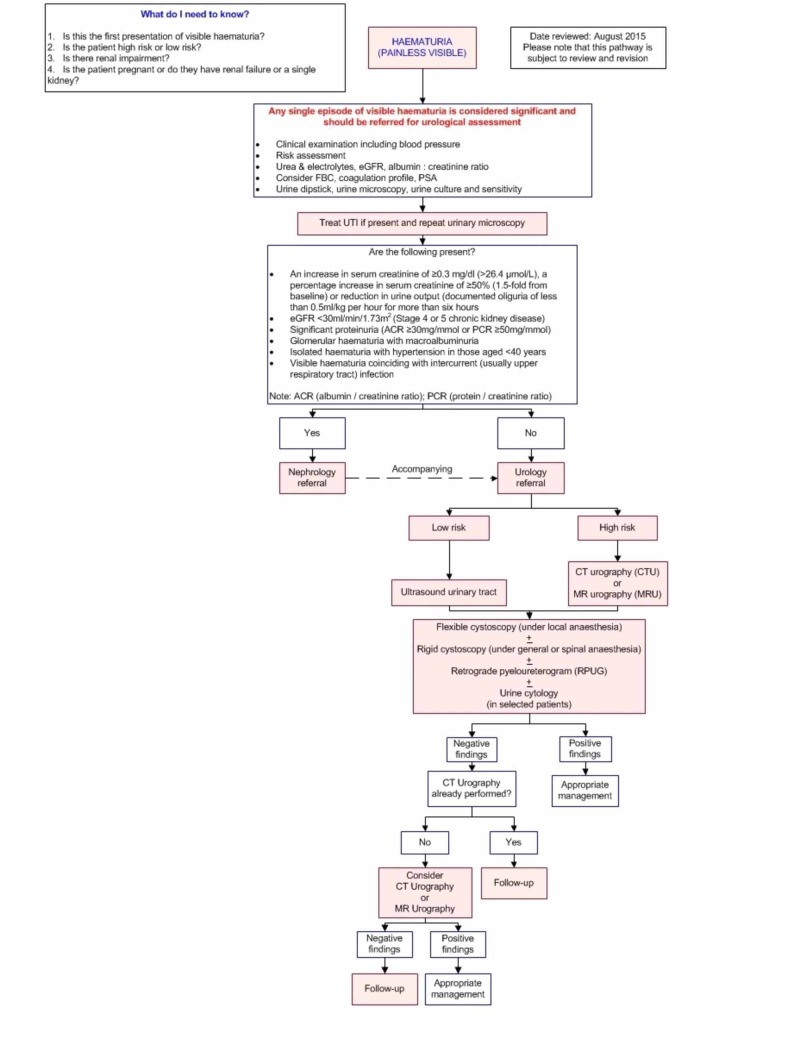
Imaging pathway This pathway provides guidance on the imaging of adult patients with unexplained painless visible haematuria. eGFR: Estimated glomerular filtration rate; FBC: Full blood count; PSA: Prostate-specific antigen; ACR: Albumin/creatinine ratio; PCR: Protein/creatinine ratio; UTI: Urinary tract infection.

A manual search to identify other relevant publications from the retrieved studies was also performed for the review. A total of 84 full-text articles were shortlisted and assessed for eligibility. Papers describing original studies, evidence-based guidelines or systematic reviews were included while paediatric and animal studies were excluded. Articles which were not in English, or had limited text were also excluded, following which 59 final articles were included and graded according to Oxford Centre for Evidence-Based Medicine Levels of Evidence [17]. The evidence-based pathways were produced and reviewed by the Diagnostic Imaging Pathways (DIP) expert editorial panel members in accordance with the usual DIP processes (http://www.imagingpathways.health.wa.gov.au/index.php/production/processes-for-creating-and-managing-content), in consultation with the editor, a urologist and a nephrologist. The final algorithm (Figure 1) which is also available on the Imaging Pathways (Online clinical decision-making tool: Dulku G. Painless Macroscopic Haematuria. Diagnostic Imaging Pathways; September 2015), provides guidance and imaging recommendations for adult patients with unexplained, painless, visible haematuria.

Our review did not include an analysis of imaging guidance for the detection of urinary tract calculi as a cause of haematuria, as typically this is associated with pain. Our focus was primarily on the imaging guidance and supporting literature for painless visible haematuria with urological malignancies as a potential cause.

Diagnostic approach and initial work-up

Consensus from the British Association of Urological Surgeons (BAUS) and the Interregional Chiefs of Urology Service (IRCUS), Kaiser Permanente, America suggested that any single episode of VH is considered significant and should be referred for urological assessment which in almost all cases will include cystoscopy [18,19].

In the interim, investigations may be carried out to identify transient, treatable causes, and arrange for primary imaging. Patients with VH and proven urinary tract infection (UTI) should still be investigated for concurrent malignancy, since malignancy commonly coexists with or acts as a nidus for an infection to propagate from [20]. Urinary tract infection should be treated prior to cystoscopy as there is a risk of uro-sepsis if cystoscopy is performed in the presence of active UTI.

The presence of VH should also not be attributed to anti-coagulant or anti-platelet therapy and these patients should also be evaluated regardless of these medications, as reports of underlying malignancy were found in 24% and 7% of patients in two separate series [9,18].

Therefore, the primary role of imaging is to identify those patients with a malignant cause of haematuria. However, the choice of imaging modality is dependent on various individual patient factors (e.g., age, risk factors for malignancy, renal function, and pregnancy) and other factors, such as local policy and practice, cost effectiveness, and availability of resources [9].

Traditionally, first-line investigations have included conventional radiography, renal ultrasound (US), and/or intravenous pyelogram (IVP) in combination with cystoscopy. The latter is important since many bleeding urinary tract lesions arise in the urinary bladder and imaging procedures are not yet conclusively proven to be as sensitive as cystoscopy in diagnosing most of them [8].

Second-line investigations have included multi-detector computed tomography urogram (MDCTU) and magnetic resonance urography (MRU), often only carried out if the first-line tests reveal an abnormality.

IRCUS has recommended that a modified CTU or IVP with concurrent renal US be performed for patients with significant haematuria. There is no need for renal tomography at intravenous urography (IVU) if a concurrent renal US is performed. This approach will reduce the exposure to ionising radiation [19].

When recommending imaging for evaluation of VH, Cowan had suggested that the type of imaging modality employed be based on a risk score with patients >40 years to undergo CT urography while patients <40 years to undergo ultrasound as the first-line imaging modality [15]. The European Society of Urogenital Radiology (ESUR) has suggested that the investigation of low-risk patients requires ultrasound and cystoscopy and high-risk patients require CTU and cystoscopy for thorough renal and urinary tract imaging [14].

Urine cytology

Urinary cytology, although controversial, is essential for the evaluation of upper urinary tract urothelial cell carcinoma (UUT-UCC) and the European Association of Urology (EAU) guidelines recommend that urinary cytology should be performed as part of the standard diagnostic work-up [12]. Urine cytology has a sensitivity of 25%, specificity of 91%, high positive predictive value but low negative predictive value [1].

However, urine cytology has a high false negative rate for the detection of malignancy and a negative cytology can never completely exclude the presence of a bladder tumour, cystoscopy is warranted in all cases.

Numerous commercially available urine tests for urine borne biomarkers include BTA TRAK, ImmunoCyt/uCyt+, CxBladder, Nuclear Matrix Protein 22 (NMP-22) and UroVysion (FISH), although these may miss a significant proportion of patients with bladder cancer particularly when its accuracy is poor for low-stage and low-grade tumours [21]. For these reasons, there was no consensus reached on the role of urine cytology and/or bladder tumour markers in the evaluation of patients with haematuria [19].

Initial imaging in low-risk patients

The ESUR had suggested that the investigation of low-risk patients requires US and cystoscopy and high-risk patients require CTU and cystoscopy for thorough renal and urinary tract imaging [14]. The sensitivity of US is, however, not sufficient to obviate the need for cystoscopy because of its lack of sensitivity in detecting small bladder tumours particularly those that are less than 0.5 cm in diameter [9, 22].

**Figure 2:**
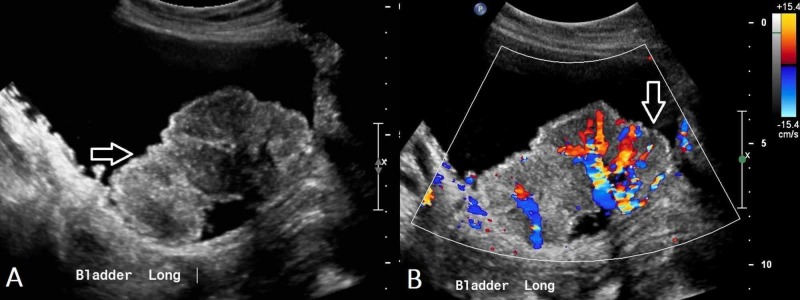
Transitional cell carcinoma of the bladder (US) (A) Ultrasound pelvis demonstrates an irregular, heterogenous within the urinary bladder with multiple smaller polypoidal lesions seen adjacent which is suggestive of urinary bladder cancer. (B) Vascularity is demonstrated within this mass.

Ultrasound (US) is important in the initial assessment of haematuria and apart from being readily available and inexpensive, it involves no exposure to ionising radiation and is especially useful in radiation-sensitive populations, such as children and pregnant or child-bearing age women. Ultrasound also permits unlimited scan planes thus allowing for good visualisation of the kidneys and urinary bladder. Additionally, Doppler studies can provide further information regarding vascularity of masses (Figure 2A, 2B) [23].

In the detection of UUT tumours, US has a variable sensitivity in detection of urothelial carcinomas, with moderate sensitivity (82%) for renal cell carcinoma detection and low sensitivity (12%) for the detection of urothelial carcinoma of the ureter [9,23]. However, when compared to IVP in the detection of abnormalities of the UUT in patients presenting with haematuria, US is more sensitive in the detection of renal and bladder tumours [9, 22, 23], with a higher sensitivity (96% versus 25%) and negative predictive value (98% versus 91%) respectively [2, 24].

**Figure 3:**
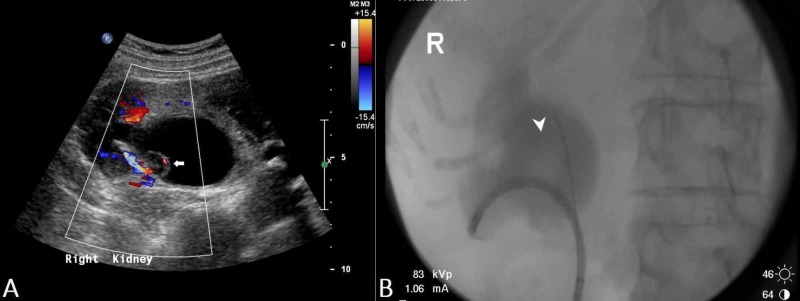
Renal transitional cell carcinoma (US and RPUG) (A) Ultrasound pelvis demonstrates a diffusely thickened proximal right ureter with a papillary lesion (arrow) seen within the right renal pelvis, and resultant right hydronephrosis. (B) This corresponds to the retrograde pyelo-ureterogram (RPUG) findings (arrow head) which is suggestive of an obstructing right upper urinary tract transitional cell carcinoma.

Furthermore, US is also useful to detect hydronephrosis and/or hydroureter which may be a sequalae of bladder tumours obstructing the vesicoureteric junction (Figure 3). Nonetheless, when compared to cross-sectional imaging such as MDCTU or MRU, US has a lower sensitivity in detecting urinary tract abnormalities.

Ultrasound is excellent in determining whether a mass is cystic or not and whether a cystic lesion is a simple cyst or minimally complicated or complicated. Lesions that are not simple cysts require further work-up with CT or MRI. Contrast-enhanced US (CEUS) is a promising alternative in the initial work-up of renal masses. The reported performance of CEUS in evaluating suspected solid or complex cystic masses suggests similar value of CEUS compared with CT. CEUS has advantages of lack of nephrotoxicity, lack of ionizing radiation, and the ability to evaluate the enhancement pattern in real time - and this may be done at the initial US attendance of the patient. However, the place of CEUS in the diagnostic algorithm needs further clarification.

Therefore, utilising US as a first-line imaging modality in the assessment of haematuria seems justifiable [23]. Given that ultrasound alone may potentially miss ureteral and urothelial lesions, further evaluation with cystoscopy is necessary while retrograde pyelo-ureterography is a useful adjunct (Figure 3) [25].

Initial imaging in high-risk patients

CT Urography

CT urography (also referred to as CT IVP though CTU is the preferable term) outperforms US, IVU, and radiography in the evaluation of renal parenchymal masses and urinary tract calculi with sensitivity and specificity for upper tract urothelial malignancies of 67-100% and 93-99%, respectively [12,26-28].

The optimum diagnostic strategy for investigating patients at high risk after excluding UTI is a combined strategy using CTU and flexible cystoscopy [12, 29]. Patients’ with lesions consistent with bladder cancer on CTU should be referred directly for rigid cystoscopy and so avoid flexible cystoscopy with an advantage of a 17% reduction in the number of flexible cystoscopies performed [30].

For all other patient categories including younger patients and patients with more benign indications and lower pre-test cancer probabilities, US is the first-line imaging modality [23, 26, 29]. CT urography examination technique modifications, consisting of limited protocols and scan phase combinations, could be utilised alternatively or complementary to the other imaging tests [14, 29].

The scan protocol should include: non-contrast scan, nephrographic (90-100 seconds post contrast bolus) and excretory (or pyelographic) phase (5-15 minutes post contrast administration) [27, 28, 31, 32]. A cortico-medullary phase is useful in selected cases, e.g., if pseudoaneurysm or pseudotumor is suspected. Routine acquisition during this phase may not be justified [33, 34]. The non-contrast images are useful to detect renal calculus. The nephrographic phase has the highest sensitivity in the detection of renal masses, and correlation with unenhanced images is required to show unequivocal enhancement. The pyelographic/excretory phase is used to assess the collecting system, ureters and bladder with the use of ureteric distention techniques such as compression, intravenous saline bolus, and diuretics all demonstrating variable results. CT urography can also detect extra-urinary disease [28].

**Figure 4:**
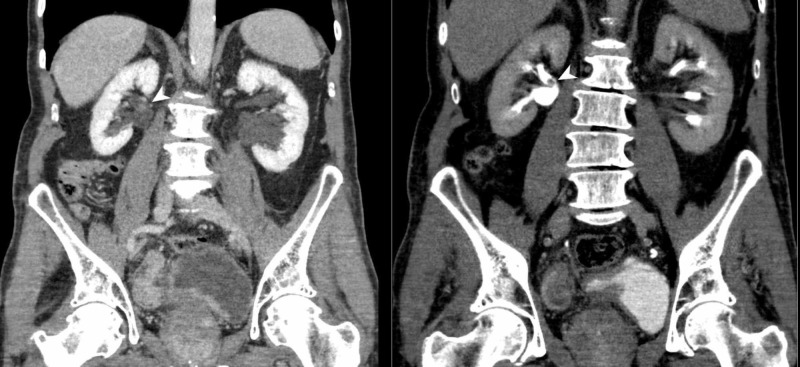
Bladder and renal TCC (CTU) CT urography (coronal reformat) demonstrates irregular urinary bladder wall thickening and also a sessile polypoid filling defect in the posterior aspect of the right renal pelvis (arrow heads). Findings are suggestive of multifocal urinary tract transitional cell carcinoma (TCC).

**Figure 5:**
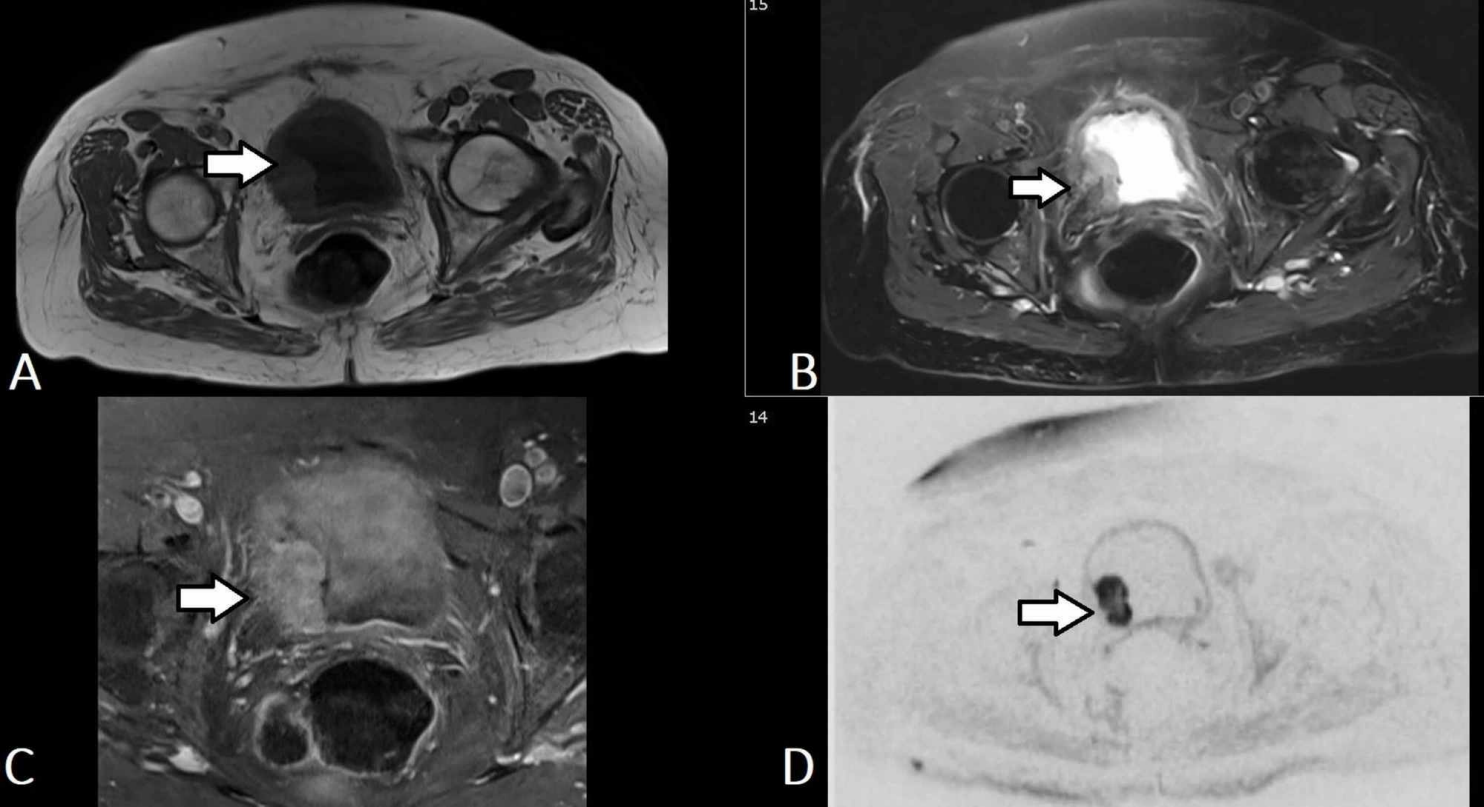
Bladder TCC (MRI) (A-D) Selected axial MR urography images of the same patient demonstrate a lobulated mass (arrow) arising from the right lateral wall of the urinary bladder which extends to the trigone and invades into the urinary bladder wall to involve the outer layer without extension into the perivesical fat. The mass is of T1 low and T2 high signal intensity. The mass demonstrates relatively homogeneous, diffuse contrast enhancement and prominent diffusion restriction (b50/400/800). The constellation of findings reflects a bladder tumour which is probably staged as T2b No Mx. TCC: Transitional cell carcinoma

Opposed to urothelial tumours, many patients with renal malignancy remain asymptomatic until late stages of the disease, given that the vast majority of renal cancers are small and detected incidentally. Additionally, renal masses rarely invade into the collecting system. Consequently, the classical triad of flank pain, haematuria, and a palpable abdominal renal mass of renal cell carcinoma (RCC) occurs in at most 6-9% of patients, and when present, it strongly suggests locally advanced disease and poor prognosis. Here, CT and MRI are each recommended for work-up and are considered equal for staging and diagnosis (Figures 4, 5) [35]. However, comparative studies between CT and high-field MRI are awaited. In the interim, MRI may be used in selected cases for problem solving. Diffusion-weighted MRI (DWI) may also be of use in lesion detection and characterisation, but systematic reviews indicate only moderate accuracy [36].

The Bosniak classification of cystic renal masses is based on imaging characteristics on contrast-enhanced CT and it is helpful in predicting the risk of malignancy and provision of guidance in management. The risk for malignancy for Bosniak 1, 2, 2F, 3, and 4 cystic lesions are 0%, 0%, 25%, 54%, and 100%, respectively [37].

CT urography can also be effective in the diagnosis of bladder tumours. However, results may differ depending on the specific population studied. In the high-risk group, unequivocal CTU results were 93% sensitive and 99% specific for detection of bladder cancer with an overall negative predictive value (NPV) of 95%. The high NPV of CTU may obviate cystoscopy in these selected patients although, cystoscopy remains the gold standard in the detection of lower urinary tract including bladder urothelial tumours, as neither IVP or MDCTU consistently have sensitivities significant enough to exclude bladder mucosal abnormalities [16,32,38,39].

Radiation doses are of a concern with MDCTU and this can be reduced by limiting the number of imaging phases through the use of dual-energy CT (DECT) or split-bolus technique [14, 40]. Additionally, (25) omission of the non-enhanced acquisition results in reduction in radiation exposure by almost 50% [41].

DECT provides information about how substances behave at different energies, the ability to generate virtual unenhanced datasets, and improved detection of iodine-containing substances on low-energy images [42-44]. Other advantages of DECT include good temporal and spatial registration and good spectral separation between high- and low-energy scans easy to equalize dose and noise [45]. A prospective study demonstrated that the single-phase DECT urography with synchronous nephrographic-excretory phase enhancement represents an accurate “all-in-one’’ approach with a radiation dose saving up to 45% compared with a standard dual-phase protocol with good opacification in 86.9% of cases and excellent or good virtual unenhanced (VUE) images in 83.3% of cases [46].

**Figure 6:**
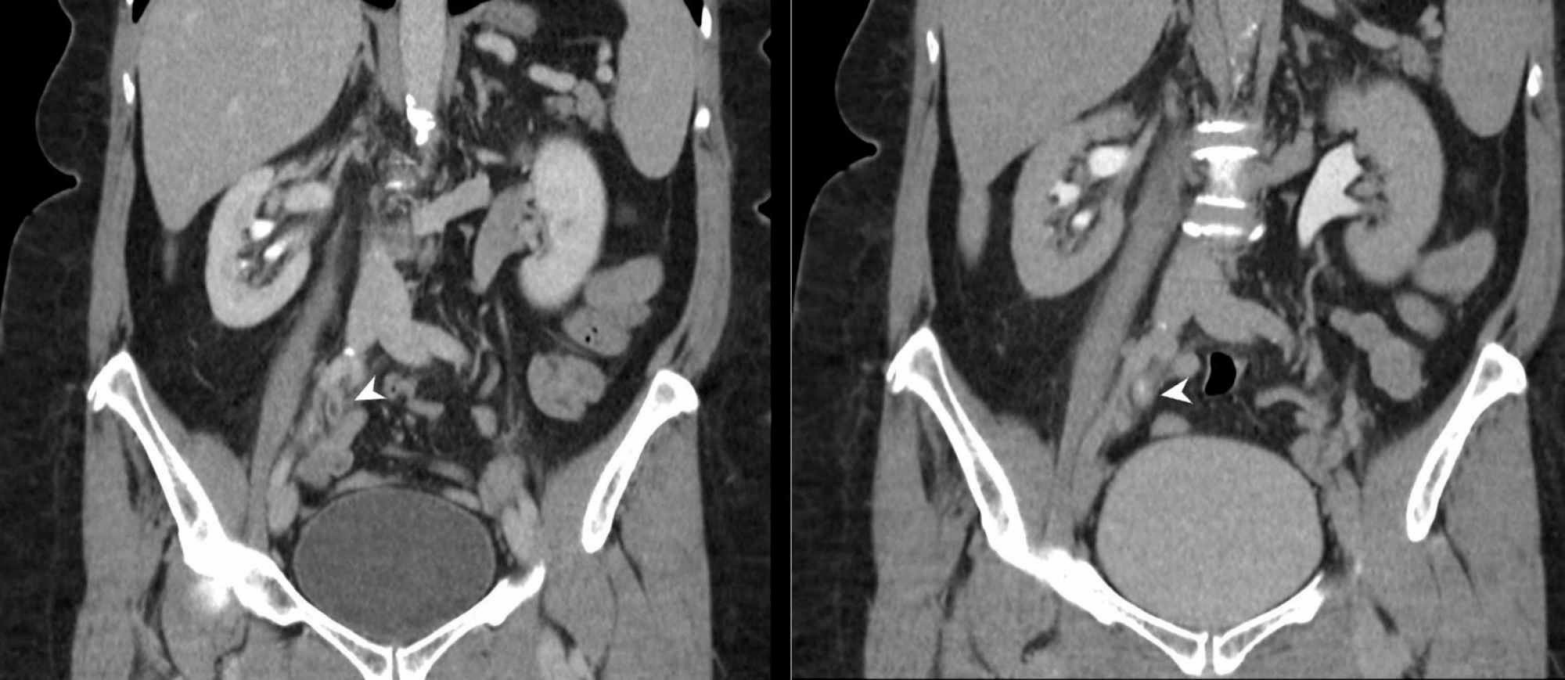
Ureteric TCC (CTU) CT urography (coronal reformats) demonstrates a 4-cm segment of circumferential urothelial thickening at the junction of the mid- and distal-right ureter (arrow heads) with abnormal urothelial enhancement. There is secondary obstruction of the right collecting system with the transition point noted at the level of abnormal urothelial thickening which is highly suspicious for a right-sided urothelial TCC. TCC: Transitional cell carcinoma

On the other hand, split-bolus MDCTU provides at least 50% opacification of the majority of UUT segments with a high sensitivity (88.9-100%), specificity (99-99.5%), and accuracy (98.5-99.5%) for the detection of upper urinary tract tumours (Figure 6) [47].

Magnetic Resonance Urography

Magnetic resonance imaging is useful in the diagnosis and staging of malignancies of the kidneys, bladder, and prostate gland and in the assessment of renal function. It serves as an alternative imaging technique for the radiation sensitive population including children and pregnant women; those requiring repeated examinations of the urinary tract and those with contraindication to iodinated contrast-media [8, 12, 28, 48, 49].

A complete MRU protocol can be used for imaging all components of the kidneys and the urinary collecting system in a single imaging session. MR urography is performed by two main methods: static-fluid urography with ultrafast T2-weighted sequences, similar to that used for magnetic resonance cholangiopancreatography (MRCP), and T1-weighted sequences of excretory urography following IV gadolinium contrast administration [28, 49].

Static-fluid MRU is preferred over excretory MRU in the imaging of patients with impaired renal function, pregnant patients and patients with ureteral obstruction particularly when there is a reasonable risk of nephrogenic systemic fibrosis [49]. However, given that the T2-weighted sequences are performed without IV contrast administration, imaging modifications are often necessary to optimise ureteric imaging.

Ureteric imaging can also be improved particularly at excretory MRU, following the administration of IV contrast material, saline solution and a diuretic to achieve a more uniform contrast distribution [49]. Although the evidence reveals that DWI only demonstrates moderate accuracy for prediction of renal malignancy, its performance as an independent test is still untested [36]. The addition of DWI to T1- and T2-weighted imaging increases the sensitivity in identifying UUT cancer with excellent inter-observer agreement. In particular, the combination of T2WI+DWI demonstrates sensitivities between 92-98% for diagnosing bladder tumours (Figure 5).

Disadvantages of MRU include its cost, availability, longer acquisition time, image degradation due to motion artefacts, lower spatial resolution and diagnostic confidence in the detection of urothelial malignancy when compared to CTU. However, the development of faster sequences and optimisation of 3-T MRI protocols are expected to address these issues [28, 36, 48, 49].

Cystoscopy

**Figure 7:**
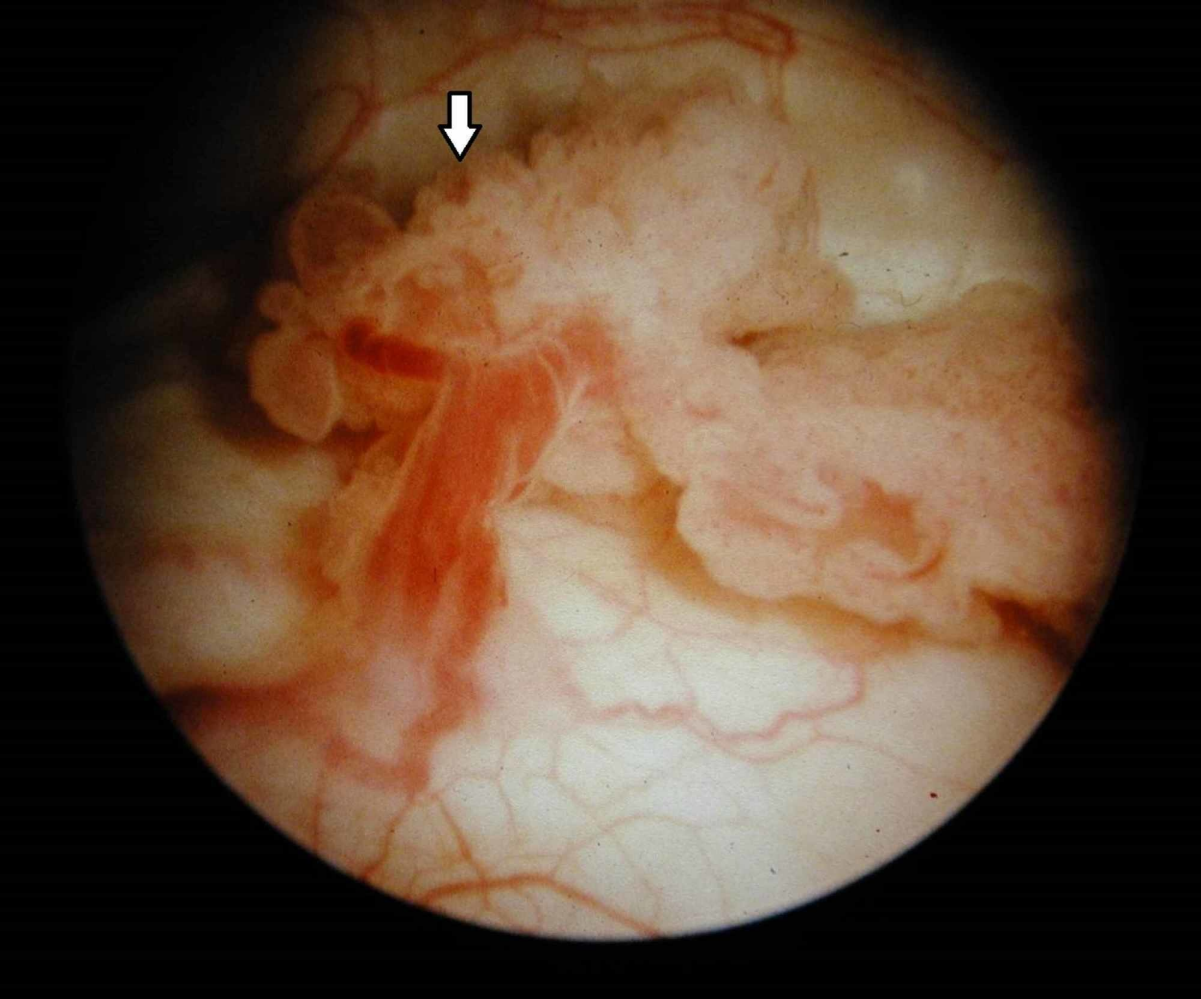
Bladder tumour (cystoscopy) Cystoscopy reveals an exophytic, cauliflower-like growth with delicate fronds and areas of ulceration in keeping with a urinary bladder malignancy (arrow).

Cystoscopy is still the method of choice for the evaluation of the urinary bladder and should not be replaced by any excretory imaging technique [29]. The recent EAU guidelines also recommend that cystoscopy should be performed to rule out concomitant bladder tumour (Figure 7) when UUT cancer is diagnosed, particularly when authors have demonstrated notable false-positive and false-negative results at CTU and MRU in the detection of bladder tumours when compared to histopathology in patients with visible haematuria [4,12]. A cystoscopy should be performed on virtually all patients with painless visible haematuria [50].

Flexible cystoscopy is simple, quick and safe procedure performed under local anaesthesia as a day case with reliable reassurance and allowing for operative patients to be diagnosed and prepared for their surgery in the same visit [11].

On the other hand, rigid cystoscopy is performed under general or spinal anaesthesia particularly when more complex additional procedures are anticipated. A retrograde pyelo-ureterogram (RPUG) can be performed at the time of rigid cystoscopy to image the upper tracts if CTU has not already been performed [25].

Other imaging studies

Retrograde Pyelo-ureterography

CT urogram has been found to have a greater diagnostic accuracy than RPUG for the detection of urothelial lesion [2, 9]. Therefore, it is justified that CTU should be used before RP, as a single, non-invasive and comprehensive test that allows simultaneous diagnosis and/or staging.

Retrograde pyelography may still be employed as a second-line investigation to further characterize filling defects detected on other modalities, non-diagnostic CTU, or in patients with renal failure or cases of contrast medium allergy. Retrograde pyelo-ureterography can only show the ureteric lumen and cannot directly depict extrinsic abnormalities (Figure 3) [2, 9, 12].

Plain Radiography of the Kidneys, Ureters and Urinary Bladder (Plain X-ray KUB)

Plain X-ray KUB currently is of little value in the investigation of patients with painless haematuria and is not used as first line image modality [2, 8, 9].

Excretory Urography/Intravenous Urography (IVU)

Intravenous urography is a good choice in evaluating urothelial and intraluminal disease but is not sufficiently sensitive for detecting renal masses <2-3 cm in size and has now been replaced by CTU for imaging the UUT [8, 22, 23]. The investigation of haematuria in high-risk patients with IVU alone is no longer advocated [2].

Its main advantage is that IVU images the entire UUT with a high degree of spatial resolution and also provides structural information as well as limited functional data. It is often the most cost-efficient test in many centres [9, 22].

However, disadvantages of IVU include lengthy acquisition time, potential hazardous contrast reactions, requiring bowel preparation and exposure to appreciable radiation load. Its relative inaccuracy is its main shortcoming and will soon be obsolete [22, 23].

Clinical follow-up

There is a risk of 1-3% of patients with a negative workup developing malignancy within three years if left unchecked [50]. The American Urological Association (AUA) best policy panel recommends that primary care physicians check for VH, new urinary symptoms, and positive cytology every six monthly for three years and if findings are consistently negative by then, follow-up may be ceased [50]. BAUS recommends re-referral to urology is necessary if there is development of VH or symptomatic non-visible haematuria (NVH) during the primary care follow-up, duration of which was not clearly defined [18, 50]. The Best Practice Advocacy Centre New Zealand have recommended annual monitoring for nephrologic causes with urine dipstick, blood pressure, eGFR and urinary albumin to creatinine ratio/protein to creatinine ratio (ACR/PCR) while haematuria persist, and annually for two year for urologic causes with urine dipstick, eGFR, urinary ACR/PCR and cytology. This initiative permits primary care follow-up to offer reassurance to patients dealing with concerns of ongoing haematuria which may be intermittent but persistent despite a negative work-up. The indication for nephrology and urology referral is summed up in Table 2 [18,19,49].

**Table 2:** Indications for referral ^† The presence of dysmorphic red blood cells, proteinuria, cellular casts, and/or renal insufficiency, or any other clinical indicator suspicious for renal parenchymal disease warrants concurrent nephrologic workup but does not preclude the need for urologic evaluation.^ ^‡ Females with asymptomatic non-visible haematuria aged <40 years do not require urology referral.^

Nephrology referral
An increase in serum creatinine of ≥0.3 mg/dl (>26.4 µmol/L), a percentage increase in serum creatinine of ≥50% (1.5-fold from baseline) or reduction in urine output (documented oliguria of less than 0.5 ml/kg per hour for more than six hours
Significant proteinuria (ACR ≥30 mg/mmol or PCR ≥50 mg/mmol) in addition to haematuria raises the suspicion of intrinsic renal disease
Glomerular haematuria with macroalbuminuria
Isolated haematuria (i.e., in the absence of significant proteinuria) with hypertension in those aged <40
Visible haematuria coinciding with intercurrent (usually upper respiratory tract) infection

Urology referral
Visible haematuria in all patient (regardless of age)
Patients with any symptomatic non-visible haematuria in the absence of UTI or other transient causes (regardless of age)
Male patients with asymptomatic non-visible haematuria
All patients with asymptomatic non-visible haematuria and other risk factors

## Conclusions

Until randomised clinical trials comparing different diagnostic modalities or strategies prospectively and outcome studies are available, recommendations based on consensus and best available evidence are nonetheless warranted to reduce the variation in haematuria management. Ultrasound remains an important diagnostic tool for the evaluation of haematuria in radiation-sensitive populations, low-risk patients and for characterizing bladder abnormalities and cystic renal lesions. Multi-detector CT urography is the most sensitive and specific test for the diagnosis of urinary tract calculi and for detecting and characterizing renal masses. Split-bolus and low-dose imaging techniques are potentially effective methods of reducing radiation dose. Magnetic resonance urography is emerging as a potentially non-invasive comprehensive imaging test for evaluating the UUT without the use of ionising radiation and thus is particularly useful in children and pregnant women. However, it is inferior to CTU in the detection of urothelial lesions. Cystoscopy is the method of choice for the evaluation of the urinary bladder, should be performed in virtually all cases of painless VH and cannot be replaced by any excretory imaging technique. This study provides an evidence-based and consensus-based review for managing painless visible haematuria in adult patients. This has the potential to reduce the incidence of unnecessary examinations by guiding clinicians towards the most appropriate diagnostic examinations and in the correct sequence.

## Appendices

Our manuscript titled “Painless visible haematuria in adults: an algorithmic approach guiding management” has not been published or submitted for publication elsewhere. Please note that only the pathway has been published on the Diagnostic Imaging Pathways website which is not a journal but a clinical decision-making tool that is used predominantly by junior doctors.
